# One-Year Frailty Transitions Among Persons With HIV Aged 70 Years or Older on Antiretroviral Treatment

**DOI:** 10.1093/ofid/ofae229

**Published:** 2024-04-26

**Authors:** Jannett Achour, Diane Abulizi, Alain Makinson, Cédric Arvieux, Fabrice Bonnet, Cécile Goujard, Oriane Lambert, Laurence Slama, Hubert Blain, Laurence Meyer, Clotilde Allavena, for the SEPTAVIH Study Group, C Goujard, S Abgrall, L Weiss, C Katlama, J-M Molina, A Cabié, F Bonnet, D Neau, A Makinson, C Allavena, V Rio, C Arvieux, D Rey, P Delobel, P Leclercq, L Slama

**Affiliations:** INSERM CESP, U1018, Paris-Saclay University, Le Kremlin-Bicêtre, France; Public Health Department, Bicêtre University Hospital, AP-HP, Le Kremlin-Bicêtre, France; INSERM CESP, U1018, Paris-Saclay University, Le Kremlin-Bicêtre, France; Infectious Diseases Department, Montpellier University Hospital, Montpellier, France; Infectious Diseases Department, Rennes University Hospital, Rennes, France; Internal Medicine Department, Bordeaux University Hospital, Bordeaux, France; INSERM CESP, U1018, Paris-Saclay University, Le Kremlin-Bicêtre, France; Internal Medicine Department, Bicêtre University Hospital, AP-HP, Le Kremlin-Bicêtre, France; INSERM CESP, U1018, Paris-Saclay University, Le Kremlin-Bicêtre, France; Infectious Diseases Department, Hôtel-Dieu Hospital, AP-HP, Paris, France; Geriatrics Department, Montpellier University Hospital, Montpellier, France; INSERM CESP, U1018, Paris-Saclay University, Le Kremlin-Bicêtre, France; Public Health Department, Bicêtre University Hospital, AP-HP, Le Kremlin-Bicêtre, France; INSERM, EA1413, Nantes, France; Infectious Diseases Department, Nantes University Hospital, Nantes, France

**Keywords:** frailty, geriatric population, HIV infection

## Abstract

**Background:**

People with HIV (PWH) are aging. Frailty is an age-related condition predictive of hospitalization and mortality. Here, we assessed the frequency and factors associated with frailty transitions at 1-year follow-up in elderly PWH.

**Methods:**

Five hundred eight PWH aged 70 years or older who were on antiretroviral treatment were included in the French multicenter SEPTAVIH study in 2019–2020. Participants were classified as robust, prefrail, or frail according to Fried frailty phenotype at baseline and at 1 year. Logistic regression models were used to evaluate socioeconomic and medical factors associated with transition between frailty states. Models were adjusted for gender, age at baseline, education, and period of HIV diagnosis (before vs after 1996).

**Results:**

Seventeen PWH died during the 1-year follow-up. Of the remaining 491 PWH (median age, 73 years), frailty status worsened for 18% of participants and improved for 14% at 1 year. Advanced age, baseline CD4+ T-cell count <350 cells/mm^3^, and type 2 diabetes were associated with transition from prefrailty to frailty (adjusted odds ratio [aOR], 1.10 per 1-year positive difference; 95% CI, 1.01–1.20; aOR, 3.05; 95% CI, 1.14–8.18; and aOR, 2.63; 95% CI, 1.05–6.57; respectively). Being female was associated with more frequent improvement from prefrailty to robustness (aOR, 2.50; 95% CI, 1.09–5.55).

**Conclusions:**

Preventing frailty in elderly PWH is a long-term problem, beginning with the early diagnosis of HIV infection and the management of comorbidities.

Worldwide, people with HIV (PWH) are aging [1]. In France, nearly 20% of PWH (33 000 individuals) will be aged 70 years or older by 2030 [2]. Frailty is an age-related condition characterized by increased vulnerability to stressors. During an acute minor event (introduction of a new drug, benign infection, etc.), a frail organism may endure a more severe, longer-lasting, and less reversible functional impairment than a nonfrail organism [3]. Fried et al. proposed a clinical screening tool for individuals age >65 years that classifies individuals as robust, prefrail, or frail according to their level of functional impairment [4]. These phenotypes are predictive of adverse health outcomes (falls, hospitalizations, institutionalizations, death), with a higher risk of negative events associated with a higher level of frailty. Frailty is a dynamic process [5]. Functional decline is commonly observed over time, but recovery may be possible with targeted interventions, such as physical exercise programs and interventions to reduce falls [6].

Most studies assessing frailty in PWH have evaluated populations with a median age between 40 and 60 years [7, 8]. The French ANRS SEPTAVIH study provided the first data on prevalence and factors associated with frailty in PWH aged ≥70 years on antiretroviral therapy (ART) [9]. Advanced age, low socioeconomic status, and multimorbidity were associated with frailty.

Identifying factors associated with frailty worsening or improvement in older PWH is fundamental to implementing and evaluating preventive strategies of functional decline in this population. Here, we assessed the frequency and factors associated with frailty transitions over 1 year in PWH aged ≥70 years on ART included in the ANRS SEPTAVIH study.

## METHODS

### Data Source

The ANRS EP66 SEPTAVIH cohort study was conducted in 16 university hospitals in France (ClinicalTrials.gov NCT03958786). Five hundred eight individuals aged ≥70 years who were infected with HIV-1, on ART for at least 12 months, and covered by French national health insurance were enrolled from May 2019 to February 2020. Individuals under legal protection or with a life expectancy <6 months were not eligible. Outpatient visits were scheduled at baseline and at 12 months. At baseline, sociodemographic factors, date of HIV diagnosis, history of clinical AIDS, comorbidities, and standard HIV and geriatric biomarkers were collected. Socioeconomic precarity was assessed using an EPICES score (Assessment of Precariousness and Health Inequalities in Health Examination Centers) ≥30.17. At baseline and at 12 months, each participant was assessed for Fried frailty phenotype, level of autonomy (Activity of Daily Living Scale and Instrumental Activity of Daily Living Scale), and cognitive and psychiatric conditions (Montreal Cognitive Assessment Scale, Center for Epidemiologic Studies Depression Scale). Hospitals collected data on incident deaths during the study period.

All participants gave written informed consent. The study design was approved by our ethics committee, CPP Ile-de-France XI (ref ID-RCB: 2018-A03100–55).

### Frailty Evaluation

The 5 components of Fried frailty score were collected by patient self-report or physical examination by a physician, as follows:

1. Shrinking: weight loss ≥5% of body weight or ≥4.5 kg in the previous year.
2. Exhaustion: self-reported by 2 questions from the Center for Epidemiologic Studies Depression Scale (“I felt that everything I did was an effort,” “I could not get going”).
3. Physical activity level: self-reported using the international physical activity questionnaire questionnaire.
4. Slowness: measured in a 4-meter walking test, adjusted for gender and height.
5. Weakness: identified as low grip strength of the dominant arm, adjusted for gender and body mass index.

The participants who suffered no impairment in any of these criteria were classified as robust. Otherwise, the participants were classified as prefrail if they had 1 or 2 functional impairments and frail if they had 3 or more functional impairments according to the Fried definition. We defined transitions between frailty states at 12-month follow-up as an improvement in functional status (from frailty to prefrailty or robustness, from prefrailty to robustness), worsening of functional status (from robustness to prefrailty or frailty, from prefrailty to frailty), or stability of Fried frailty phenotype.

We determined the Fried phenotype for each participant at baseline and at 12 months. If 1 or more of the 5 criteria was missing for a given patient, the Fried score was considered missing.

### Missing Data Handling

Missing data for Fried score were handled by multiple imputation by chained equations (mice R package) under the missing-at-random assumption, meaning that the probability that a value is missing depends only on the observed values [10]. Incomplete Fried scores at baseline and at the 12-month visit and missing Fried scores for lost-to-follow-up participants were completed in the same model. We used the highest prevalence of missingness among the 5 components of Fried score at baseline or 12 months to determine the number of data sets to impute [11]. Twenty-two data sets were imputed. The variables used in the imputation model are reported in Supplementary Table 1. Missing criteria for Fried score were imputed by predictive mean matching; then a Fried score was calculated for each participant. The analyses described below were done separately for each imputed data set, then pooled according to Rubin's rules [12].

### Statistical Analyses

Participants who died during the 1-year follow-up were not included in the analysis of frailty transitions. We compared the sociodemographic and medical characteristics of participants with a complete Fried score and participants with an incomplete or missing Fried score at baseline and at 12 months. Categorical variables were compared using the chi-square or Fisher test. Continuous variables were compared using the Kruskal-Wallis test.

We calculated the mean frequency of robust, prefrail, and frail participants at baseline and at 12 months over the 22 imputations.

Logistic regression models were used to evaluate factors associated with frailty transition at 12 months according to Fried frailty phenotype at baseline. We considered the following outcomes: (1) deterioration of robust PWH toward prefrailty or frailty vs stability of robustness over 1 year, (2) improvement of prefrail PWH toward robustness vs stability of prefrailty over 1 year, (3) deterioration of prefrail PWH toward frailty vs stability of prefrailty over 1 year, and (4) improvement of frail PWH toward prefrailty or robustness vs stability of frailty over 1 year.

To study factors associated with frailty transitions in robust and prefrail participants, models were adjusted for potential confounders according to the literature: gender, age at baseline, level of education (college degree vs less education), and period of HIV infection diagnosis (before vs after July 15, 1996, right after the 11th International Conference on AIDS in Vancouver, which approved the use of protease inhibitors in combined antiretroviral therapy) [13–15]. Age as a continuous variable respected the linearity hypothesis with the logit of dependent variables. We excluded any collinearity between the variables introduced into the multivariable models by analyzing the cross-tabulated data for the dichotomous variables and assessing the variance inflation factor [16]. Considering the small size of the frail population at baseline, we only performed univariable analysis to study factors associated with improvement in this group.

Analyses were performed using SAS (version 9.3) and R (version 4.2.1).

## RESULTS

### Characteristics at Baseline

**Figure 1.**
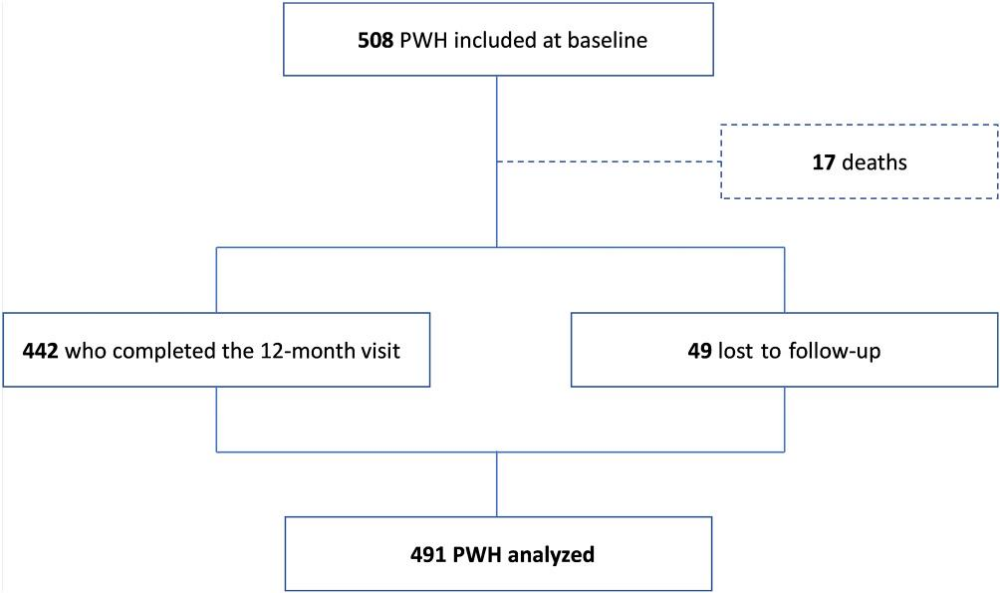
Flowchart.

Four hundred ninety-one participants were included in the analysis, of whom 49 were lost to follow-up during the first year (Figure 1). Ninety-four point three percent of participants had an undetectable viral load at baseline. The median age at enrollment was 73 years (95% CI, 71.6–77.0), and 81.5% of participants were men. Most participants were born in Western Europe (81.0%) and did not suffer from socioeconomic precarity (65.8%). Forty point seven percent of participants had a college degree. The median known duration of HIV infection was 22.7 years (95% CI, 15.5–28.1). Thirty-seven point six percent of PWH were aged 30–50 years at the time of HIV diagnosis, and 10.8% were diagnosed after the age of 65. Forty-seven point five percent of PWH had been diagnosed before the widespread use of protease inhibitors in 1996. A clinical AIDS stage was reported in 27.7% of the study population, of whom 53% were diagnosed with HIV infection before 1996. Most PWH were autonomous at baseline in their activities of daily living (ADL score ≥5 in 99.0% of cases and IADL score ≥6 in 91.4% of cases).

### Patterns of Missing Data for Multiple Imputation

At baseline, there were no missing data for sociodemographic factors, HIV infection factors, and comorbidities. Three hundred eighty-eight (79.0%) and 324 (66.0%) participants had a complete Fried score at baseline and at the 12-month visit, respectively. The most frequent missing criterion was self-reported exhaustion, both at baseline (10.8%) and at the 12-month visit (22.8%, half of which came from participants who were lost to follow-up) (Supplementary Figure 1). Comparison of the baseline characteristics of participants with incomplete and complete Fried scores are shown in Supplementary Tables 2–5. No statistically significant differences were observed between these 2 groups with regards to socioeconomic and medical factors at baseline. Participants lost to follow-up (n = 49) more often had a baseline MOCA score <26 (*P* = .03), but the distribution of Fried frailty phenotypes was similar to that of participants not lost to follow-up. Participants with an incomplete Fried score at 1 year, including PWH lost to follow-up, were older (*P* < .01) and more often had a baseline CD4+ T-cell count <350/mm^3^ (*P* < .01) and a MOCA score <26 (*P* < .01) than participants with a complete Fried score. Age, CD4+ T-cell count, and MOCA score were included in the imputation model.

### Frailty Prevalence

**Figure 2.**
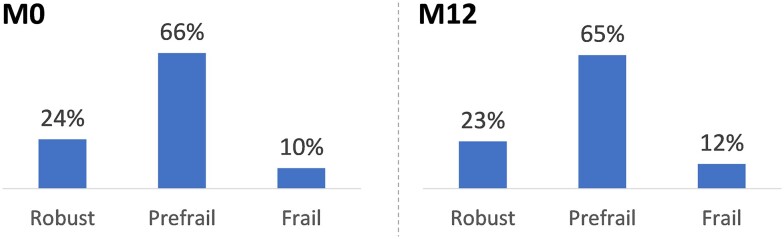
Prevalence of frailty according to Fried Frailty phenotype at baseline and the 12-month visit (n = 491). Prevalence rates were calculated as the mean frequencies of Fried frailty phenotypes in the 22 imputed data sets.

Prevalence rates of prefrailty and frailty at baseline and 12 months remained stable (Figure 2).

### Characteristics of Participants According to the Fried Frailty Phenotype at Baseline

**Figure 3.**
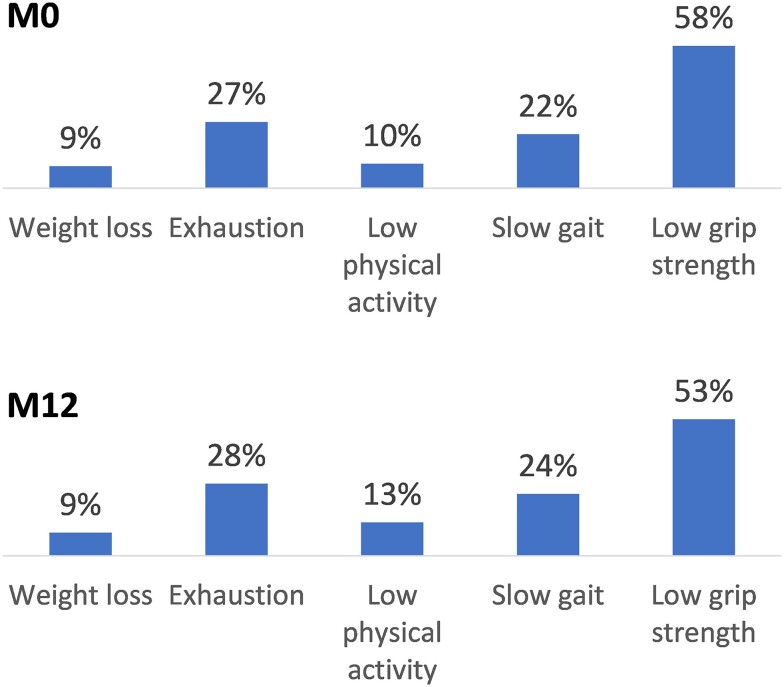
Frequency of frailty criteria according to Fried frailty phenotype in prefrail and frail participants at baseline (M0) and at the 12-month visit (M12). Prevalence rates were calculated as the mean frequencies for a given criterion in the 22 imputed data sets.

At baseline, prefrail and frail PWH were significantly older and more often precarious than robust PWH (Table 1) and more frequently had depressive symptoms and cognitive impairment. Frail PWH were significantly more likely to have been born in Sub-Saharan Africa and to have hypertension or type 2 diabetes than robust PWH. They also more frequently had a body mass index <22 kg/m^2^, the threshold of underweight in a geriatric population. The most frequent criteria associated with prefrailty or frailty were low grip strength and exhaustion, followed by slow gait (Figure 3).

**Table 1.** Participants’ Characteristics According to Their Fried Frailty Phenotype at Baseline (n = 491)

Variables, No. (%) or Median [IQR]	Robust (n = 119)	Prefrail (n = 324)	*P* Value^a^	Frail (n = 48)	*P* Value^b^
Male	93 (78.2)	267 (82.4)	.33	40 (83.3)	.45
Age, y	72.7 [71.0–74.8]	73.9 [71.7–77.2]	<.01	76.5 [73.3–81.5]	<.01
BMI <22 kg/m^2^	19 (16.0)	65 (20.1)	.36	15 (31.3)	.03
BMI >30 kg/m^2^	11 (9.2)	44 (13.6)	.24	8 (16.7)	.16
College education level	55 (46.2)	124 (38.3)	.12	21 (43.8)	.79
Deprived socioeconomic status^c^	27 (22.7)	111 (34.3)	.03	30 (62.5)	<.01
Born in Sub-Saharan Africa	15 (12.6)	46 (14.2)	.71	14 (29.2)	.01
Duration of known HIV infection, y	21.9 [15.8–26.9]	22.7 [15.5–27.7]	.68	24.3 [14.6–30.4]	.45
HIV diagnosis before 1996	54 (45.4)	153 (47.2)	.78	26 (54.2)	.37
History of clinical AIDS	30 (25.2)	87 (26.9)	.69	19 (39.6)	.07
Baseline CD4+ T-cell count <350/mm^3^	17 (14.3)	46 (14.2)	.97	7 (14.6)	.83
Multimorbidity (≥2 comorbidities)^d^	94 (79.0)	268 (82.7)	.62	42 (87.5)	.70
Current smoker^e^	9 (7.6)	30 (9.3)	.46	9 (18.7)	.09
High blood pressure^f^	72 (60.5)	216 (66.7)	.25	39 (81.2)	.01
Type 2 diabetes^g^	23 (19.3)	64 (19.8)	.88	17 (35.4)	.03
Chronic kidney disease^h^	46 (38.7)	125 (38.6)	.99	26 (54.2)	.07
Depressive symptoms^i^	4 (3.4)	74 (22.8)	<.01	20 (41.7)	<.01
Cognitive impairment^j^	50 (42.0)	199 (61.4)	<.01	34 (70.8)	<.01

The numbers of robust (n = 119), prefrail (n = 324), and frail (n = 48) participants were calculated as the mean prevalence of each phenotype in the 22 imputed data sets. Data are presented as the mean frequency (percentage) across the imputed data sets for categorical variables and as the median [interquartile range] across the imputed data sets for continuous variables. Characteristics of prefrail and frail participants were compared, respectively, with characteristics of robust participants by univariable multinomial regression and pooled on imputed data sets according to Rubin's rules.

Abbreviations: BMI, body mass index; CKD-EPI, Chronic Kidney Disease Epidemiology Collaboration; IQR, interquartile range; PWH, people with HIV.

^a^ *P* value for tests comparing prefrail PWH with robust PWH.

^b^ *P* value for tests comparing frail PWH with robust PWH.

^c^Assessed by an EPICE (Assessment of Precariousness and Health Inequalities in Health Examination Centers) score ≥30.17.

^d^High blood pressure, type 2 diabetes, angina/myocardial infarction, stroke and associated disorders, peripheral artery disease, dyslipidemia, chronic kidney disease, chronic respiratory disease, osteoporosis, cancer in medical record (except cervical cancer, non-Hodgkin lymphoma, Kaposi, and basal/spinoid skin cancers).

^e^At least 1 cigarette per day.

^f^Systolic blood pressure ≥140 mmHg or diastolic blood pressure ≥90 mmHg or previous diagnosis in medical record.

^g^Glycosylated hemoglobin >7% or previous clinical diagnosis in medical record.

^h^Estimated filtration rate <60 mL/min/1.73 m² (using the CKD-EPI equation) or diagnosis in medical record.

^i^CES-D (Center for Epidemiologic Studies–Depression Scale) score ≥17 for men and ≥23 for women (French standards).

^j^MOCA (Montreal Cognitive Assessment Scale) score <26.

### Transitions Between Frailty States

Sixty-eight percent of participants kept the same phenotype at 1 year, while 18% saw their functional status deteriorate and 14% improved.

**Figure 4.**
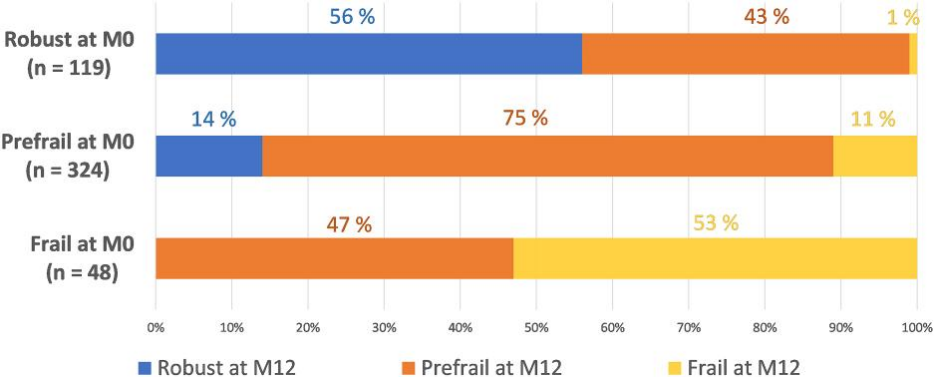
Evolution of Fried frailty phenotypes at 12 months according to frailty status at baseline (n = 491). Numbers of robust (n = 119), prefrail (n = 324), and frail (n = 48) participants were calculated as the mean prevalence of each phenotype in the 22 imputed data sets. We reported the mean frequencies of each type of frailty transition in the imputed data sets.

Among the robust PWH at baseline, 43% worsened to prefrailty, and 1% worsened directly to frailty (Figure 4). This progression was mainly related to a deterioration of grip strength (from 0% at baseline to 38.5% at M12) and self-reported exhaustion (from 0% at baseline to 31.4% at M12) (Supplementary Table 6). Sociodemographic and HIV infection factors were not associated with the transition from robustness to prefrailty or frailty (Table 2). Robust PWH with a history of hypertension were surprisingly more likely to remain robust at 12 months than robust PWH without a history of hypertension.

**Table 2.** Factors Associated With the Evolution of Robust and Prefrail Participants at 12 Months

	Robust at M0			Prefrail at M0					
	Worsening to Prefrailty or Frailty (n = 52) vs Stability (n = 66)			Worsening to Frailty (n = 35) vs Stability (n = 244)			Improvement to Robustness (n = 44) vs Stability (n = 244)		
	OR^a^	95% CI	*P*	OR^b^	95% CI	*P*	OR^b^	95% CI	*P*
Crude OR^c^									
Male	0.50	[0.20–1.27]	.15	0.81	[0.29–2.32]	.71	0.47	[0.21–1.03]	**.06**
Age^e^	0.97	[0.85–1.10]	.64	1.10	[1.01–1.20]	**.02**	0.93	[0.84–1.03]	.16
College education	0.86	[0.39–1.92]	.71	0.95	[0.39–2.29]	.91	1.57	[0.77–3.20]	.21
Socioeconomic deprivation^f^	1.40	[0.56–3.49]	.47	1.63	[0.71–3.77]	.25	1.13	[0.52–2.44]	.76
Born in Sub-Saharan Africa	0.68	[0.19–2.43]	.55	1.47	[0.51–4.26]	.48	1.28	[0.50–3.31]	.61
HIV diagnosis <1996	0.54	[0.24–1.21]	.14	1.21	[0.54–2.71]	.64	1.87	[0.91–3.84]	.09
AIDS stage	0.57	[0.22–1.48]	.24	1.38	[0.57–3.33]	.47	1.23	[0.58–2.62]	.58
Baseline CD4+ count <350/mm^3^	1.18	[0.35–3.98]	.79	2.76	[1.05–7.27]	**.04**	1.39	[0.51–3.84]	.52
Multimorbidity^g^	0.65	[0.26–1.63]	.36	1.34	[0.43–4.17]	.61	1.09	[0.41–2.88]	.86
Hypertension^h^	0.44	[0.20–0.99]	**.05**	0.86	[0.36–2.03]	.72	0.94	[0.45–1.93]	.86
Type 2 diabetes^i^	0.95	[0.36–2.53]	.92	2.44	[1.01–5.91]	**.05**	0.88	[0.33–2.37]	.80
Chronic kidney disease^j^	0.60	[0.27–1.36]	.22	1.43	[0.64–3.19]	.37	0.69	[0.30–1.58]	.38
Adjusted OR^d^									
Male	0.56	[0.21–1.44]	.22	0.81	[0.28–2.37]	.70	0.40	[0.18–0.92]	**.03**
Age^e^	0.97	[0.84–1.10]	.60	1.10	[1.01–1.20]	**.03**	0.93	[0.84–1.03]	.18
College education	0.83	[0.36–1.91]	.65	1.01	[0.39–2.57]	.98	1.55	[0.73–3.28]	.25
Socioeconomic deprivation^f^	1.27	[0.49–3.31]	.61	1.51	[0.63–3.61]	.35	1.27	[0.56–2.89]	.56
Born in Sub-Saharan Africa	0.58	[0.16–2.16]	.41	1.38	[0.55–2.94]	.58	1.45	[0.54–3.87]	.46
HIV diagnosis <1996	0.56	[0.25–1.35]	.21	1.22	[0.53–2.78]	.64	1.77	[0.84–3.70]	.13
AIDS stage	0.57	[0.21–1.52]	.26	1.33	[0.54–3.29]	.53	1.30	[0.60–2.82]	.51
Baseline CD4+ count <350/mm^3^	1.06	[0.30–3.69]	.93	3.05	[1.14–8.18]	**.03**	1.54	[0.54–4.40]	.41
Multimorbidity^g^	0.69	[0.26–1.82]	.45	1.17	[0.37–3.68]	.79	0.96	[0.35–2.67]	.95
Hypertension^h^	0.45	[0.20–1.02]	**.06**	0.80	[0.33–1.98]	.63	1.07	[0.51–2.26]	.86
Type 2 diabetes^i^	1.05	[0.38–2.89]	.92	2.63	[1.05–6.57]	**.04**	0.89	[0.32–2.47]	.82
Chronic kidney disease^j^	0.53	[0.22–1.26]	.15	1.21	[0.53–2.78]	.65	0.76	[0.32–1.80]	.53

Abbreviations: CKD-EPI, Chronic Kidney Disease Epidemiology Collaboration; OR, odds ratio; PWH, people with HIV.

^a^Reference for odds ratio: robust PWH at baseline that remained robust at 12 months.

^b^Reference for odds ratio: prefrail PWH at baseline that remained prefrail at 12 months.

^c^Crude model.

^d^Each OR is adjusted for gender, age at baseline, college education level, and period of HIV diagnosis (before vs after 1996).

^e^Age is centered on 70 years; odds ratio is for a positive difference of 1 year of age.

^f^Assessed by an EPICE (Assessment of Precariousness and Health Inequalities in Health Examination Centers) score ≥30.17.

^g^High blood pressure, type 2 diabetes, angina/myocardial infarction, stroke and associated disorders, peripheral artery disease, dyslipidemia, chronic kidney disease, chronic respiratory disease, osteoporosis, cancer in medical record (except cervical cancer, non-Hodgkin lymphoma, Kaposi, and basal/spinoid skin cancers).

^h^Systolic blood pressure ≥140 mmHg or diastolic blood pressure ≥90 mmHg or previous diagnosis in medical record.

^i^Glycosylated hemoglobin >7% or previous clinical diagnosis in medical record.

^j^Estimated filtration rate <60 mL/min/1.73 m² (using the CKD-EPI equation) or diagnosis in medical record.

Seventy-five percent of the prefrail PWH at baseline remained prefrail at 12 months, 14% reversed to robustness, and 11% worsened to frailty. Of the prefrail at enrollment who became frail at 12 months, 79.3% already had a poor hand grip at baseline (Supplementary Table 6). Fried scores increased due to low physical activity (from 8.6% at baseline to 62.1% at M12), weight loss (from 2.9% at baseline to 31.4% at M12), self-reported exhaustion (from 34.3% at baseline to 72.4% at M12), and decline in walking speed (from 45.7% at baseline to 74.3% at M12). A positive difference of 1 year of age, a CD4+ T-cell count <350 cells/mm^3^, and type 2 diabetes were significantly associated with worsening to frailty, in both crude and multivariable analyses considering gender, age at baseline, college education level, and period of HIV diagnosis (adjusted odds ratio [aOR], 1.10; 95% CI, 1.01–1.20; aOR, 3.05; 95% CI, 1.14–8.18; and aOR, 2.63; 95% CI, 1.05–6.57; respectively).

Fourteen percent of prefrail PWH at baseline recovered to robustness at 12 months. They improved mainly in hand grip (from 66.7% at baseline to 0% at M12) and exhaustion (from 27.1% at baseline to 0% at M12); they also improved in physical activity (from 9.1% to 0% at M12) (Supplementary Table 7). Men had a lower risk of improvement in the crude analysis and after adjustment for age at baseline, education, and period of HIV diagnosis (aOR, 0.40; 95% CI, 0.18–0.92).

Forty-seven percent of the frail PWH at baseline improved to prefrailty at 12 months, mainly through improvement in physical activity, with a prevalence of low physical activity of 21.7% at 12 months compared with 73.9% at baseline, and through improvement in self-reported exhaustion, with a prevalence of exhaustion of 26.0% at 12 months compared with 78.3% at baseline (Supplementary Table 7). None of the frail PWH recovered to robustness. No sociodemographic or medical factors were associated with improvement at 12 months.

We compared the crude odds ratios of frailty transitions estimated with multiple imputation with those obtained with complete cases. Overall, crude odds ratios were similar in both analyses (Supplementary Table 8). The variance of the estimated regression coefficients was smaller in the pooled analysis of imputed data sets than in complete cases due to the larger population size in the imputed data.

The 17 participants who died during the study period did not differ significantly from the other participants in terms of age at baseline, duration of known HIV infection, AIDS status, baseline CD4+ T-cell count, or multimorbidity. However, deceased participants were more frequently socioeconomically disadvantaged than nondeceased participants (64.7% vs 34.2%, respectively). The frequency of mortality was higher among frail participants at baseline than among robust participants (7.7% vs 1.7%, respectively) (Supplementary Table 9).

## DISCUSSION

The SEPTAVIH study provides original longitudinal data on frailty in PWH aged 70 years and older. Over 1 year, stability of functional status was more frequent than deterioration or improvement. Direct transition from robustness to frailty was rare. No participants recovered from frailty to robustness. Advanced age, baseline CD4+ T-cell count <350/mm^3^, and type 2 diabetes were associated with transition from prefrailty to frailty. Men recovered less often from frailty to prefrailty than women.

Deterioration of functional status was slightly more frequent than improvement. Lorenzo-López et al. found similar results in a 1-year study of a population-based cohort aged 65 years and older [17]. A longer follow-up of elderly PWH will likely reveal a higher frequency of progression to frailty [18, 19]. Phenotype changes most often occurred between adjacent frailty states, suggesting a gradual process that enables early detection of new functional impairment and the implementation of remediation strategies to limit or reverse the deterioration of functional status over time.

We found a relationship between immunological status, as measured by CD4 cell counts, and the onset of frailty in prefrail PWH over 1 year. The DUNEDIN and POLSENIOR cohorts, which included HIV-uninfected young adults and elderly, respectively, also established an association between aging of the immune system and functional decline [20–22]. Immunosenescence may occur with chronologic age or prematurely in PWH [23]. In PWH, this accelerated immunosenescence may promote excessive frailty compared with HIV-uninfected people of the same age [24]. Molina-Pinelo et al. suggested that low CD4+ T-cell count may be a marker of premature aging of the immune system in PWH on ART [25]. Our results are also consistent with the analysis of middle-aged men with HIV in the MACS cohort, in which a low CD4+ T-cell count was strongly predictive of frailty over time [15].

Physiological knowledge gives insight on the association of type 2 diabetes with transition from prefrailty to frailty over 1 year. Yanase et al. suggested a positive feedback between type 2 diabetes and frailty [26]. In this model, glucose-mediated cellular oxidative stress, mitochondrial dysfunction, chronic low-grade inflammation, and hormonal dysfunction would promote deterioration of muscle and nerve functions and loss of executive functions that are characteristics of frailty. Sarcopenia is the most important physical factor associated with frailty [27]. Exercise training should be encouraged, particularly in diabetic PWH, to prevent age-related physical decline [28]. The association of hypertension with maintenance of robustness at 12 months was unexpected [4]. One year of follow-up may be too short to observe incident hypertension-related events such as stroke or heart failure that are risk factors for frailty [4, 29].

We found no significant association between the period of HIV diagnosis (before vs after 1996) or the history of clinical AIDS and the progression to frailty. This contrasts with the results of the MACS cohort, in which an HIV diagnosis before 1996 or having AIDS was associated with frailty in middle-aged men [15]. In the MACS cohort, PWH may have suffered from an excess of morbidity linked to suboptimal therapeutic management in the late 90s to early 2000s, compared with PWH followed in a more recent period in SEPTAVIH. Besides, the limited sample or a selective survival effect in our study may explain these discrepancies.

We previously found that participants in the SEPTAVIH study were similar to PWH of the same age followed in French hospitals at the same time in terms of country of birth, duration of known HIV infection, immunologic status, and virologic status [9]. Thus, the findings of the SEPTAVIH study can probably be extrapolated to French PWH of the same age on ART.

Our study has certain limitations. First, we had no information on the time each participant spent in the categories of the Fried score before the study, whereas this duration may influence later patterns of frailty transition. Second, using multiple imputation requires the missing-at-random assumption. This hypothesis seems appropriate as some observed variables (college education level, CD4+ T-cell count, and chronic cognitive disorders) were associated with missing data for Fried score. Third, we cannot exclude that some of the changes in frailty phenotypes were due to variability. This should be considered in future intervention trials, which must include a control arm. Nevertheless, in our study factors associated with worsening frailty phenotypes differed from those associated with improved phenotypes, suggesting that part of the transitions in frailty phenotypes were attributable to real change in health. Finally, we cannot exclude a possible impact of the coronavirus disease 2019 pandemic on the frailty components of the Fried score. In particular, the level of physical activity and the feeling of exhaustion (secondary to isolation and possible depression) may have increased during this period. Of note, however, all assessments at M12 were made after the end of the first lockdown period in France. Fatigue and level of physical activity measured according to the Fried score only concerned the 7 days preceding the medical visit at M12, and therefore did not coincide with the strict lockdown period for most participants. Among the 17 patients who died before M12 and were not included in the analysis of frailty transitions, only 2 died of COVID-19 during the study period.

## CONCLUSIONS

The frailty state of PWH aged 70 years or older can vary rapidly over the course of a year. Importantly, short-term improvement of functional capacities is possible, offering opportunity for prevention and rehabilitation strategies. Apart from constitutive risk factors such as advanced age and gender, our study highlights modifiable risk factors, such as type 2 diabetes, for transition to frailty. Promoting early HIV diagnosis and high adherence to ART to maintain high levels of CD4 could be associated with less evolution to frail phenotypes. Long-term follow-up of SEPTAVIH participants will enable us to better identify frailty risk factors. Assessing frailty in elderly PWH, for which the literature is scarce, is important to identify intervention levers specific to HIV infection.

## Supplementary Data

Supplementary materials are available at *Open Forum Infectious Diseases* online. Consisting of data provided by the authors to benefit the reader, the posted materials are not copyedited and are the sole responsibility of the authors, so questions or comments should be addressed to the corresponding author.

## Supplementary Material

ofae229_Supplementary_Data
