# Supplementary material for: One-Year Frailty Transitions Among Persons With HIV Aged 70 Years or Older on Antiretroviral Treatment
Source: Open Forum Infect Dis. 2024 Apr 26;11(7):ofae229. doi: 10.1093/ofid/ofae229 (PMC11222971; doi:10.1093/ofid/ofae229)
Supplement: ofae229_Supplementary_Data [file ofae229_supplementary_data.docx]

**One-year frailty transitions among persons living with HIV aged 70 years or more on ART**

Jannett Achour^1^, Diane Abulizi^1^, Alain Makinson^2^, Cédric Arvieux^3^, Fabrice Bonnet^4^, Cécile Goujard^5^, Oriane Lambert^1^, Laurence Slama^6^, Hubert Blain^7^, Laurence Meyer^1, 8^, Clotilde Allavena^9, 10^, and the SEPTAVIH Study Group

*Author information*

^1^INSERM CESP, U1018, Paris-Saclay University Le Kremlin-Bicêtre, France

^2^Montpellier University Hospital, Infectious Diseases Department, Montpellier, France

^3^Rennes University Hospital, Infectious Diseases Department, Rennes, France

^4^Bordeaux University Hospital, Internal Medicine Department, Bordeaux, France

^5^Bicêtre University Hospital, Internal Medicine Department, AP-HP, Le Kremlin-Bicêtre, France

^6^Hôtel-Dieu Hospital, Infectious Diseases Department, AP-HP, Paris, France

^7^Montpellier University Hospital, Geriatrics Department, Montpellier, France

^8^Bicêtre University Hospital, Public Health Department, AP-HP, Le Kremlin-Bicêtre, France

^9^INSERM, EA1413, Nantes, France

^10^Nantes University Hospital, Infectious Diseases Department, Nantes, France

**Appendix**

1. **Supplementary table 1:** Variables included in the imputation model.
2. **Supplementary table 2:** Comparison of the baseline characteristics of participants with complete data and participants with missing data for the Fried score at baseline
3. **Supplementary table 3:** Comparison of the baseline characteristics of participants with complete data and participants with missing data for Fried score at twelve-months.
4. **Supplementary table 4:** Comparison of the baseline characteristics of participants who came to the twelve-months visit and participants lost-to-follow-up.
5. **Supplementary table 5:** Comparison of the Fried frailty phenotypes at baseline of participants who came to the twelve-months visit and participants lost to follow-up.
6. **Supplementary table 6:** Prevalence of frailty criteria of robust and prefrail participants at baseline who worsened their functional capacities at twelve-months.
7. **Supplementary table 7:** Prevalence of frailty criteria of prefrail and frail participants at baseline who improved their functional capacities at twelve-months.
8. **Supplementary table 8:** Factors associated with the evolution of robust and prefrail participants over one year, on complete cases, in univariable analyses.
9. **Supplementary table 9:** Comparison of the vital status at twelve-months according to the Fried frailty phenotype at baseline.
10. **Supplementary figure 1:** Frequency of missing data for Fried score at baseline and at twelve-months follow-up.

**Supplementary table 1. Variables included in the imputation model.**

| Type | Variable |
| --- | --- |
| Fried score at baseline | - Weight measured at baseline - Self-reported weight one-year before inclusion in SEPTAVIH - Height measured at baseline - Self-reported evaluation of two questions measuring exhaustion: never to a few times a week/ frequently or always (questions: “I felt that everything I did was an effort”, “I could not get going”). - *Metabolic* *Equivalent of Task* from self-reported level of physical activity - Walking speed - Grip strength on the left hand - Grip strength on the right hand - Dominant hand |
| Fried score at twelve-months visit | - Weight measured at twelve-months visit - Self-reported evaluation of two questions measuring exhaustion: never to a few times a week/ frequently or always (questions: “I felt that everything I did was an effort”, “I could not get going”). - *Metabolic* *Equivalent of Task* from self-reported level of physical activity - Walking speed - Grip strength on the left hand - Grip strength on the right hand |
| Sociodemographic characteristics | - Age at baseline (years, continuous variable) - Gender - Born in sub-Saharan Africa (yes/ no) - Socio-economic status (EPICE score, continuous variable) - College education level (yes/ no) |
| HIV infection | - Diagnostic of HIV infection before the 15^th^ of July, 1996 (yes /no) - Duration of known HIV infection (years, continuous variable) - AIDS status (yes/ no) - CD4+ T-cell count (cell/mm^3^, continuous variable) at baseline |
| Comorbidities | - Number of comorbidity among hypertension, stroke, angina/ myocardial infarction, arteritis, type 2 diabetes, dyslipidemia, chronic kidney disease, chronic obstructive pulmonary disease/ emphysema, osteoporosis, non-haematological cancer (except basal or spinoid skin cancer, cervical cancer, Kaposi sarcoma) - Current smoker *versus* previous or no smoker - Hypertension - Type 2 diabetes - Chronic kidney disease |
| Cognitive and psychiatric conditions at baseline | - *Montreal Cognitive Assessment* score - *Center for Epidemiologic Studies Depression* score |

In the imputation model, we included the following variables: 1) all variables required to calculate the Fried score, 2) sociodemographic or medical variables we assumed *a priori* to be associated with missing data, 3) variables statistically associated with missing data for the Fried score and 4) variables we assumed to be associated with one-year frailty transitions in PLWH.

**Supplementary table 2. Comparison of the baseline characteristics of participants with complete data and participants with missing data for the Fried score at baseline (n = 491).**

| Variables, n (%) or median [IQR] | Complete data for Fried score  (n = 388) | Missing data for Fried score  (n = 103) | p-value |
| --- | --- | --- | --- |
| Male | 320 (82.5) | 79 (76.7) | 0.23 |
| Age (years) | 73.5  [71.5 ; 77.0] | 74.1  [71.9 ; 77.3] | 0.50 |
| College education level | 154 (39.7) | 46 (44.7) | 0.45 |
| Deprived socioeconomic status^a^ | 237 (61.1) | 54 (52.4) | 0.42 |
| Born in sub-Saharan Africa | 58 (14.9) | 17 (16.5) | 0.81 |
| Duration of known HIV infection (years) | 23.1  [15.8 ; 27.7] | 21.1  [14.4 ; 28.6] | 0.35 |
| HIV diagnosis before 1996 | 189 (48.7) | 43 (41.7) | 0.24 |
| History of clinical AIDS | 112 (28.9) | 23 (22.3) | 0.25 |
| Baseline CD4+ T-cell count < 350/mm^3^ | 55 (14.2) | 14 (13.6) | 1.00 |
| Undetectable HIV viral load | 366 (94.3) | 97 (94.2) | 1.00 |
| Multimorbidity (>2 comorbidities)^b^ | 307 (79.1) | 80 (77.7) | 0.73 |
| Current smoker^c^ | 160 (41.2) | 34 (33.0) | 0.15 |
| High blood pressure^d^ | 260 (67.0) | 68 (66.0) | 0.94 |
| Type 2 diabetes^e^ | 82 (21.1) | 21 (20.4) | 0.96 |
| Chronic kidney disease^f^ | 158 (40.7) | 38 (36.9) | 0.55 |
| Chronic cognitive disorders^g^ | 212 (54.6) | 58 (56.3) | 0.68 |

Qualitative variable : number, percentage, Chi square test

Quantitative variable : median, interquartiles, Kruskal-Wallis test

^a^Assessed by an EPICE (Assessment of precariousness and health inequalities in health examination centers) score ≥ 30.17.

^b^diseases: high blood pressure, type 2 diabetes, angina/myocardial infarction, stroke and associated disorders, peripheral artery disease, dyslipidemia, chronic kidney disease, chronic respiratory disease, osteoporosis, cancer in medical record (except cervical cancer, non-Hodgkin lymphoma, Kaposi, and basal/spinoid skin cancers).

^c^At least one cigarette per day.

^d^Systolic blood pressure ≥140 mmHg or diastolic blood pressure ≥ 90 mmHg or previous diagnosis in medical record.

^e^Glycosylated hemoglobin > 7% or previous clinical diagnosis in medical record.

^f^Estimated filtration rate < 60 mL/min/1.73 m² (using the CKD-EPI equation) or diagnosis in medical record.

^g^MOCA (*Montreal Cognitive Assessment Scale*) score < 26.

**Supplementary table 3. Comparison of the baseline characteristics of participants with complete data and participants with missing data for Fried score at twelve-months (n = 491).**

| Variables, n (%) or median [IQR] | Complete data for Fried score  (n = 329) | Missing data for Fried score  (n = 162) | p-value |
| --- | --- | --- | --- |
| Male | 267 (81.2) | 132 (81.5) | 1.00 |
| Age (years) | 73.3  [71.5 ; 76.5] | 74.4  [72.2 ; 78.1] | **<0.01** |
| College education level | 135 (41.0) | 65 (40.1) | 0.95 |
| Deprived socioeconomic status^a^ | 100 (30.4) | 62 (38.3) | 0.13 |
| Born in sub-Saharan Africa | 46 (14.0) | 29 (17.9) | 0.32 |
| Duration of known HIV infection (years) |  |  |  |
| HIV diagnosis before 1996 | 163 (49.5) | 69 (42.6) | 0.19 |
| History of clinical AIDS | 85 (25.8) | 50 (30.9) | 0.28 |
| Baseline CD4+ T-cell count < 350/mm^3^ | 36 (10.9) | 33 (20.4) | **<0.01** |
| Undetectable HIV viral load | 314 (95.4) | 149 (92.0) | 0.24 |
| Multimorbidity (>2 comorbidities)^b^ | 261 (79.3) | 126 (77.8) | 0.83 |
| Current smoker^c^ | 133 (40.4) | 61 (37.7) | 0.60 |
| High blood pressure^d^ | 225 (68.4) | 103 (63.6) | 0.34 |
| Type 2 diabetes^e^ | 71 (21.6) | 32 (19.8) | 0.73 |
| Chronic kidney disease^f^ | 128 (38.9) | 68 (42.0) | 0.58 |
| Chronic cognitive disorders^g^ | 158 (48.0) | 112 (69.1) | **<0.01** |

The category of participants with missing data for Fried score at twelve-months follow-up (n = 162) include lost-to-follow-up patients (n= 49).

Qualitative variable : number, percentage, Chi square test

Quantitative variable : median, interquartiles, Kruskal-Wallis test

^a^Assessed by an EPICE (Assessment of precariousness and health inequalities in health examination centers) score ≥ 30.17.

^b^diseases: high blood pressure, type 2 diabetes, angina/myocardial infarction, stroke and associated disorders, peripheral artery disease, dyslipidemia, chronic kidney disease, chronic respiratory disease, osteoporosis, cancer in medical record (except cervical cancer, non-Hodgkin lymphoma, Kaposi, and basal/spinoid skin cancers).

^c^At least one cigarette per day.

^d^Systolic blood pressure ≥140 mmHg or diastolic blood pressure ≥ 90 mmHg or previous diagnosis in medical record.

^e^Glycosylated hemoglobin > 7% or previous clinical diagnosis in medical record.

^f^Estimated filtration rate < 60 mL/min/1.73 m² (using the CKD-EPI equation) or diagnosis in medical record.

^g^MOCA (*Montreal Cognitive Assessment Scale*) score < 26.

**Supplementary table 4. Comparison of the baseline characteristics of participants who came to the twelve-months visit and participants lost to follow-up (n= 491).**

| Variables, n (%) or median [IQR] | Participants who came to the 12-months visit  (n = 442) | Participants lost to follow-up  (n = 49) | p-value |
| --- | --- | --- | --- |
| Male | 358 (81.0) | 41 (83.7) | 0**.**79 |
| Age (years) | 73.7  [71.6 ; 77.0] | 73.0  [71.7 ; 77.1] | 0.65 |
| College education level | 177 (40.0) | 23 (46.9) | 0.38 |
| Deprived socioeconomic status^a^ | 142 (32.1) | 20 (40.8) | 0.26 |
| Born in sub-Saharan Africa | 71 (16.1) | 4 (8.2) | 0.21 |
| Duration of known HIV infection (years) | 23.0  [15.6 ; 27.8] | 20.8  [14.7 ; 27.8] | 0.37 |
| HIV diagnosis before 1996 | 212 (48.0) | 20 (40.8) | 0.50 |
| History of clinical AIDS | 120 (27.1) | 15 (30.6) | 0.67 |
| Baseline CD4+ T-cell count < 350/mm^3^ | 61 (13.8) | 8 (16.3) | 0.81 |
| Undetectable HIV viral load | 418 (94.6) | 45 (91.8) | 0.45 |
| Multimorbidity (>2 comorbidities)^b^ | 352 (79.6) | 35 (71.4) | 0.26 |
| Current smoker^c^ | 173 (39.1) | 21 (42.9) | 0.73 |
| High blood pressure^d^ | 301 (68.1) | 27 (55.1) | 0.09 |
| Type 2 diabetes^e^ | 95 (21.5) | 8 (16.3) | 0.53 |
| Chronic kidney disease^f^ | 177 (40.0) | 19 (38.8) | 0.99 |
| Chronic cognitive disorders^g^ | 236 (53.4) | 34 (69.4) | **0.03** |

Qualitative variable : number, percentage, Chi square test

Quantitative variable : median, interquartiles, Kruskal-Wallis test

^a^Assessed by an EPICE (Assessment of precariousness and health inequalities in health examination centers) score ≥ 30.17.

^b^high blood pressure, type 2 diabetes, angina/myocardial infarction, stroke and associated disorders, peripheral artery disease, dyslipidemia, chronic kidney disease, chronic respiratory disease, osteoporosis, cancer in medical record (except cervical cancer, non-Hodgkin lymphoma, Kaposi, and basal/spinoid skin cancers).

^c^At least one cigarette per day.

^d^Systolic blood pressure ≥140 mmHg or diastolic blood pressure ≥ 90 mmHg or previous diagnosis in medical record.

^e^Glycosylated hemoglobin > 7% or previous clinical diagnosis in medical record.

^f^Estimated filtration rate < 60 mL/min/1.73 m² (using the CKD-EPI equation) or diagnosis in medical record.

^g^MOCA (*Montreal Cognitive Assessment Scale*) score < 26.

**Supplementary table 5. Comparison of the Fried frailty phenotypes at baseline of participants who came to the twelve-months visit and participants lost to follow-up (n= 491).**

| Variables,  n (%) | Participants who came to the 12-months visit  (n = 442) | Participants  lost to follow-up  (n = 49) | p-value* |
| --- | --- | --- | --- |
| Robust | 108 (24.4) | 11 (22.4) | 0.95 |
| Prefrail | 291 (65.8) | 33 (67.3) |  |
| Frail | 43 (9.7) | 5 (10.2) |  |

Data obtained with multiple imputation for the Fried score. The number of each category of participants corresponds to the mean prevalence of the corresponding category over the twenty-two imputed datasets.

*pooled Chi square

**Supplementary table 6. Prevalence of frailty criteria of robust and prefrail participants at baseline who worsened their functional capacities at twelve-months.**

| Variables,  n (%) | Robust PLWH at baseline who became prefrail or frail at M12  (n = 52) | | Prefrail PLWH at baseline who became frail at M12  (n = 35) | |
| --- | --- | --- | --- | --- |
|  | **Present at M0** | **Present at M12** | **Present at M0** | **Present at M12** |
| Weight loss | 0 (0) | 10 (19.2) | 1 (2.9) | 11 (31.4) |
| Exhaustion | 0 (0) | 15 (28.8) | 12 (34.3) | 22 (62.9) |
| Low physical activity | 0 (0) | 10 (19.2) | 3 (8.6) | 18 (51.4) |
| Slow gait | 0 (0) | 10 (19.2) | 16 (45.7) | 26 (74.3) |
| Low grip strength | 0 (0) | 20 (38.5) | 25 (71.4) | 28 (80.0) |

We reported the mean frequencies of each frailty criteria over the twenty-two imputed datasets.

**Supplementary table 7. Prevalence of frailty criteria of prefrail and frail participants at baseline who improved their functional capacities at twelve-months.**

| Variables,  n (%) | Prefrail PLWH at baseline who became robust at M12 (n = 44) | | Frail PLWH at baseline who became robust or prefrail at M12 (n = 23) | |
| --- | --- | --- | --- | --- |
|  | **Present at M0** | **Present at M12** | **Present at M0** | **Present at M12** |
| Weight loss | 9 (20.5) | 0 (0) | 11 (47.8) | 0 (0) |
| Exhaustion | 12 (27.3) | 0 (0) | 18 (78.3) | 6 (26.0) |
| Low physical activity | 4 (9.1) | 0 (0) | 17 (73.9) | 5 (21.7) |
| Slow gait | 5 (11.4) | 0 (0) | 20 (87.0) | 10 (43.5) |
| Low grip strength | 25 (56.8) | 0 (0) | 18 (78.3) | 15 (65.2) |

We reported the mean frequencies of each frailty criteria over the twenty-two imputed datasets.

**Supplementary table 8. Factors associated with the evolution of robust and prefrail participants over one year, on complete cases, in univariable analyses.**

|  | **Robust at M0** | | | **Prefrail at M0** | | | | | |
| --- | --- | --- | --- | --- | --- | --- | --- | --- | --- |
|  | **Progression to prefrailty or frailty (n=32)**  ***vs.* stability (n=44)** | | | **Progression to frailty (n=13)**  ***vs.* stability (n=131)** | | | **Improvement to robustness (n=28) *vs.* stability (n=131)** | | |
|  | **OR*** | **IC95%** | **p** | **OR**** | **IC95%** | **p** | **OR**** | **IC95%** | **p** |
| Male | 0.35 | [0.11; 1.09] | 0.07 | 0.44 | [0.13; 1.51] | 0.19 | 0.45 | [0.17 ; 1.21] | 0.11 |
| Age^†^ | 0.93 | [0.79; 1.09] | 0.37 | 1.15 | [1.03; 1.29] | **0.02** | 0.96 | [0.85; 1.07] | 0.43 |
| College education | 1.02 | [0.41; 2.56] | 0.96 | 1.13 | [0.39 ; 3.32] | 0.82 | 1.26 | [0.55; 2.93] | 0.58 |
| Socioeconomic deprivation ^a^ | 1.71 | [0.54; 5.41] | 0.36 | 2.77 | [0.90; 8.53] | 0.08 | 1.66 | [0.71; 3.90] | 0.24 |
| Born in sub-Saharan Africa | 0.47 | [0.11; 1.91] | 0.29 | 3.10 | [0.87; 11.03] | 0.08 | 1.39 | [0.42; 4.60] | 0.59 |
| HIV Diagnosis < 1996 | 0.52 | [0.21; 1.31] | 0.17 | 0.83 | [0.29; 2.35] | 0.72 | 2.42 | [1.02; 5.75] | **0.05** |
| AIDS stage | 0.35 | [0.10; 1.21] | 0.10 | 1.45 | [0.47 ; 4.48] | 0.52 | 1.53 | [0.63; 3.72] | 0.35 |
| Baseline CD4+ count <350/mm^3^ | 0.90 | [0.23; 3.51] | 0.88 | 4.56 | [1.22; 17.03] | **0.02** | 2.52 | [0.70; 9.05] | 0.16 |
| Multimorbidityb | 0.37 | [0.12; 1.10] | 0.07 | 1.37 | [0.29; 6.50] | 0.69 | 0.76 | [0.28; 2.10] | 0.60 |
| Hypertension^c^ | 0.38 | [0.14; 0.98] | **0.05** | 0.73 | [0.25; 2.16] | 0.57 | 1.14 | [0.46; 2.80] | 0.78 |
| Type 2 diabetes^d^ | 1.09 | [0.36; 3.31] | 0.88 | 2.04 | [0.64; 6.48] | 0.23 | 0.94 | [0.33; 2.73] | 0.91 |
| Chronic kidney disease^e^ | 0.60 | [0.23; 1.56] | 0.29 | 2.03 | [0.71; 5.78] | 0.19 | 0.77 | [0.32; 1.83] | 0.55 |

OR: odds ratio. IC95%: 95% confidence interval.

*reference for odds-ratio: robust PLWH at baseline that remained robust at twelve months.

** reference for odds-ratio: prefrail PLWH at baseline that remained prefrail at twelve months.

^a^Assessed by an EPICE (Assessment of precariousness and health inequalities in health examination centers) score ≥ 30.17.

^b^Number of comorbidities was calculated based on the following diseases: high blood pressure, type 2 diabetes, angina/myocardial infarction, stroke and associated disorders, peripheral artery disease, dyslipidemia, chronic kidney disease, chronic respiratory disease, osteoporosis, cancer in medical record (except cervical cancer, non-Hodgkin lymphoma, Kaposi, and basal/spinoid skin cancers).

^c^Systolic blood pressure ≥140 mmHg or diastolic blood pressure ≥ 90 mmHg or previous diagnosis in medical record.

^d^Glycosylated hemoglobin > 7% or previous clinical diagnosis in medical record.

^e^Estimated filtration rate < 60 mL/min/1.73 m² (using the CKD-EPI equation) or diagnosis in medical record.

**Supplementary table 9. Comparison of the vital status at twelve-months according to the Fried frailty phenotype at baseline.**

| Variables,  n (%) | Robust  (n = 121) | Prefrail  (n = 335) | Frail  (n = 52) | p** |
| --- | --- | --- | --- | --- |
| Dead | 2 (1.7) | 11 (3.3) | 4 (7.7) | 0.10 |
| Alive | 119 (98.3) | 324 (96.7) | 48 (92.3) |  |

We used a new imputation model to complete missing data for Fried score. This model contains variables necessary to calculate Fried score at baseline only (not at twelve-months), and the same socio-demographic and medical variables detailed in supplementary table 1.

For imputed data, the number of each category of participants was derived from the mean prevalence of the corresponding category over the twenty-two imputed datasets.

*Fisher’s test/ **Pooled chi square test with Yates correction

**Supplementary figure 1. Frequency of missing data for Fried score at baseline (M0) and at twelve-months follow-up (M12).**
